# Estimating particle size and velocity from fluorescence pulses: A practical validation study of flow cytometry signals analysis

**DOI:** 10.1371/journal.pone.0348292

**Published:** 2026-05-18

**Authors:** Megan A. Catterton, Matthew DiSalvo, Paul N. Patrone, Gregory A. Cooksey

**Affiliations:** 1 Microsystems and Nanotechnology Division, National Institute of Standards and Technology (NIST), Gaithersburg, Maryland, United States of America; 2 Applied and Computational Math Division, National Institute of Standards and Technology (NIST), Maryland, Gaithersburg, United States of America; Italian Institute of Technology, ITALY

## Abstract

When particles cross optimal measurement regions in a flow cytometer, they generate time-resolved signals (pulses) that contain valuable information. However, this information is lost when signals are reduced to scalars such as area, width, and height. Because flow cytometers convolve particle properties with the laser profile and geometric factors of the optical interrogation region, it is in theory possible to estimate the particle diameter, velocity, and brightness (effectively its fluorophore concentration or scattering factors) from the time-resolved data alone. Here, we independently vary the velocity, size and dye concentration to validate their unique contributions to signal shape under flow conditions in which particle trajectories are well controlled. Through a series of flow rate variations on microspheres of known size and fluorophore concentrations, we study the magnitude of changes in time-resolved signals and the impact on estimation of particle properties. The method was applied to cells labeled with a DNA binding dye, which is frequently used to classify cells whether cells are in pre- (G0/G1; 1x DNA label) or post mitosis (G2; 2x DNA label) phase of the cell cycle. Using our signals analysis, we found a 1.26-fold increase in the apparent diameter from G0/G1 to G2 populations. This shift corresponds to an approximate doubling of the volume of a sphere, which is consistent with literature and microscopy data. Overall, this work validates that fluorescence pulses acquired in flow cytometry can be used to estimate and remove sources of uncertainty (e.g., due to velocity changes) and to extract information about the size, velocity, and biomarker concentrations of particles and cells.

## Introduction

Flow cytometers have the capacity to make high-throughput measurements of the optical properties of single cells and particles and relate these data to biological properties such as size, composition, and identity [1,2]. Through various optical paths, flow cytometers collect signals that arise from light-matter interactions (such as scattering or fluorescence) as objects pass through illuminated volumes. These signals are inherently time-varying, but traditional processing simplifies each measurement down to scalar values, e.g., the signals’ integrated area, pulse height (in intensity) and pulse width (in time). While these properties of the data can be computed efficiently using electronics for analog signals processing [3,4], they remain insufficient to probe important phenomena such as cell deformation or to resolve interdependencies between size and velocity.

Modern digital acquisition cards, processor speeds, and storage devices are more than capable of obtaining and storing full signal pulses (traces) [4–8], but novel analysis methods are needed to extract useful information from these more expansive data. A few approaches have utilized the full time-series from cytometry pulses. Early work showed measurements of rise time or pulse widths can be used to estimate particle size [4,6,7]. Digitized pulses have also been used for classification of similar populations [6,8].

More recently, we developed a model [6] that quantifies variations in the fluorescence pulses in terms of linear transformations associated with physically meaningful effects such as: 1) a pulse widening or narrowing due to variation in particle velocities and 2) a signal amplification or attenuation associated with particle size and fluorophore concentration. Notably, these transformations, which are performed in the frequency domain, could be combined with mathematical optimization to estimate the relative sizes and instantaneous velocities of particles. To achieve this, however, it is necessary to select a reference signal against which other time series are compared. In our original study, we demonstrated that this analysis yields accurate size and velocity estimates when applied to well-characterized calibration particles (i.e., fluorescent beads). However, it remains an open question if this method extends to real-world flow cytometry experiments.

This work seeks to validate, extend, and address the practical utility of our spectral time-series analysis (STA) for particle sizing and velocimetry in flow cytometry. Various realistic scenarios are constructed and tested, including 1) random *versus* guided selection of the reference signal; 2) robustness to changes in instrument flow rates; 3) handling of multiple sub-populations with different densities; and 4) tunability to either fixed or variable concentration of the fluorescent label. To experimentally control each scenario, we make use of our recently reported microfluidic serial flow cytometer capable of precise flow focusing with minimal fluorescence measurement variations (≈ 0.1% coefficient of variation (CV) of velocity and < 2% per-particle fluorescence measurement uncertainty) [9]. The serial cytometer also provides additional validation of per-particle velocities, which can be calculated from the time-of-flights between serial measurement regions. Ultimately, we test the method using a challenging sample consisting of a heterogenous mixture of biological cells with labeled nuclei. In doing so, we both validate the applicability of STA to cellular flow cytometry and demonstrate a novel measurement of the relative size change of the nuclear compartment in G1 and G2 cells based on the stained DNA [4,5,7,10–15].

## Materials and methods

Certain equipment, instruments, software, or materials are identified in this paper in order to specify the experimental procedure adequately. Such identification is not intended to imply recommendation or endorsement of any product or service by NIST, nor is it intended to imply that the materials or equipment identified are necessarily the best available for the purpose.

### Microcytometer fabrication

Microfluidic cytometers were fabricated using previously published methods [9]. Briefly, two micromolded layers of poly(dimenthylsiloxane) (PDMS) were aligned and bonded face-to-face to form a microdevice as previously described. Light-blocking channels were filled with black PDMS and optical fibers were set within light-transmitting channels using UV-curable optical adhesive, as previously detailed [9,16].

### Serial cytometer configuration and signals analysis

The microfluidic cytometers used here contained at least 2 measurement regions. Each measurement region consisted of an excitation waveguide that delivered an approximately uniform, flat-top profile across the flow channel. A fluorescence emission collection waveguide was positioned on the upstream side of the excitation waveguide, and a transmission collection waveguide was placed on the opposite side of the flow channel across from the excitation waveguide.

Signals from the serial cytometer were digitized, processed, and logged in real-time by a data acquisition card (8 analog inputs, 16-bit resolution, 2 × 10^6^ sample s^-1^) using previously established methods (for more details, see DiSalvo et al. [9]). Fluorescence emission from particles excited by a laser with a wavelength of 395 nm or 488 nm (diode laser, 0.5% stability over 8 h, 0.2% RMS noise) was filtered by a quadpass filter (passbands from 415 nm to 473nm, 499 nm to 547nm, 572 nm to 616 nm, and a long pass above 656 nm) and detected by amplified photomultiplier tubes (PMTs) (extended red multialkali photocathode with wavelength range 230 nm to 920 nm and radiant sensitivity of 78 mA/W at 630 nm). As previously reported, the transmitted light, which was used to observe changes in light intensity as particles traversed the measurement region, was directly detected with silicon photodetectors (± 2% uniformity, ± 0.5% linearity) [9]. Briefly, pulses with intensities above a threshold magnitude were extracted from the raw data and logged as.h5 files using the HDF5 file format [9]. Simultaneously, data for each pulse’s magnitude (“height”), full width at half maximum (“width”), and integrated area (“area”) were calculated and stored. Pulses were then matched between regions using the previously reported time-of-flight prediction method [9]. The matched data were then used to calculate the time-averaged velocities of particles based on the separation distance and time of flight between the measurement regions. To directly compare signals from particles with different velocities, the time axis of a particle was normalized by multiplying time by the ratio of each particle’s velocity to the mean velocity of the fastest particles.

### Sample preparation

Solutions of beads with manufacturer-specified fluorescent dye equivalence (MESF; see S1 Table in S1 File) were prepared in Dulbecco’s modified phosphate buffered saline (DPBS) with 0.1% (by volume) Triton X-100 (Thermo Fisher Scientific). Neutral buoyancy of beads was achieved by adding a saturated solution of sodium chloride (≈ 6.14 mol/L) to an approximate volume fraction of 35%. Jurkat cells were fixed in 4% paraformaldehyde for 15 min, rinsed, and stained in 5 mg/mL Hoechst 33342 (Thermo Fisher Scientific) diluted in 1 × DPBS solution for 30 min at room temperature. The cells were then rinsed 3 times and resuspended in a solution of Iodixanol (16% by volume to achieve neutral buoyancy) in DPBS.

### Serial cytometer experiments

Syringe pumps (30 µm/min minimum pulse-free actuation speed) were used to control flow rates. All sheath and sample fluids were loaded in glass syringes and connected to their respective inlets by tubing as previously described [9]. Total flow rates were varied throughout this study, but to establish stable inertial focusing of particles, the syringe pump flow rates maintained ratios of 1: 3: 3: 2: 15 representing the sample: up: down: left: right portions of the channel cross section, respectively (see S2 Table in S1 File for flow rates and velocities used during the experiment). The particle-based Reynold’s number was calculated as previously described [9,17].

Measurements began after particle transit times between measurement regions stabilized, which was determined by the coefficient of variation (CV; one standard deviation divided by the mean) of particle velocities reaching a consistent minimum value.

### Bead comparison

Relative concentrations of dyes within beads of different mean equivalent fluorescein (MEFL) were calculated from:


$$\begin{array}{c} Concentration=\frac{MEFL}{{\frac{\text{4}}{\text{3}}\pi r}^{\text{3}}}, \end{array}$$ (1)

where *MEFL* is obtained from the product specification sheet for the bead and *r* is the mean radius of the bead also according to the specification sheet (see SI for a table of MEFL values).

When measurements were collected during changing flow conditions, data were first gated on velocity to group analyses by similar flow rates. Then bead populations were manually gated on bivariate plots produced by dimensionality reduction of fluorescence and transmission using principal component analysis (PCA) of log-transformed measurements. See S1 Fig in S1 File for an example of the gating.

### Application of the spectral time-series analysis

First, sub-populations of interest were selected from the gated data. Next, background was subtracted from the event pulses in the selected sub-populations. Pulses were centered in a 2 ms window based on the midpoint between the rising and falling of the signal’s half maximum intensity. Traces of insufficient length were padded with randomly sampled background. To correct signal intensities for different fluorophore concentrations, we multiplied raw fluorescence data by a correction factor (CF) defined as


$$\begin{array}{c} CF=\;\frac{Median\;height\;population}{Median\;height\;reference\;population}. \end{array}$$

Relative velocity and relative size (which can be scaled to calibration particles or by orthogonal measurements with absolute sizing capabilities, e.g., microscope or Coulter counter) were extracted using a previously developed optimization algorithm that iteratively solves for the particle properties [6]. The main idea behind this approach is to first select a reference signal associated with a particle having unknown radius $R_{\text{0}}$ and velocity $v_{\text{0}}$. Next, we model how changes in velocity, size, and fluorophore concentration deform the reference signal into a measured *test signal*, and vice versa. As discussed in Patrone et al., [6], it is convenient to do this analysis in Fourier space, since a characteristic time series has on the order of a thousand points while the pulse in the frequency domain only has 10–20 modes above the noise floor. Thus, the Fourier representation is two orders of magnitude smaller in dimension than its time domain representation.

**Fig 1:**
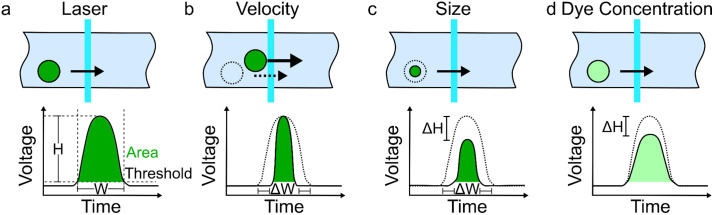
Diagrams of convolution of laser profile with an example particle and the resulting voltage versus time. **a)** Shows an example particle and its resulting voltage versus time. For purposes of illustration, this can be considered a reference signal. Measurements of pulse height (H), width (W) and area (green under the curve) are highlighted. **b)** Changes in the velocity primarily affects the width of the signal. These effects are characterized by the matrix $\frac{{𝐯}_{\text{m}}}{{\text{v}}_{\text{0}}}𝐕({\text{v}}_{\text{m}},{\text{v}}_{\text{0}})$ in Eq. (1). **c)** Difference in size affects both the height and width. These effects are characterized by the matrix $𝐒({\text{R}}_{\text{m}},{\text{R}}_{\text{0}})$ in Eq. (1). **d)** Difference in the dye concentration primarily affects the height of the pulse, which is characterized by the factor of ${𝐜}_{\text{m}}/{\text{c}}_{\text{0}}$ in Eq. (1). The main idea behind our signals analysis is to determine the set of physical parameters (particle size and speed) that can be used to transform the measured signal into the reference by minimizing the difference $\Lambda {\hat{\text{f}}}_{\text{m}}-{\hat{\text{f}}}_{\text{0}}$; see Eq. (1). Note that differences in size can only be detected if the particle radius is on the same order of magnitude as the width of the laser profile.

With this in mind, the Fourier representation of changes due to velocity, size, and concentration can be represented as


$$\begin{array}{c} \Lambda {\hat{f}}_{m}=\;{\hat{f}}_{\text{0}} \end{array}$$

where ${\hat{f}}_{\text{0}}$ is the Fourier transform of the fluorescence time-series $f_{\text{0}}(t)$ of the reference particle, ${\hat{f}}_{m}$ is the corresponding Fourier transform of the measured (or test) signal, and Λ is the transformation matrix that converts one signal into the other. While details are presented in [6], we previously showed that Λ can be expressed in the form


$$\begin{array}{c} \Lambda =\frac{v_{m}c_{\text{0}}}{v_{\text{0}}c_{m}}S\left(R_{m},R_{\text{0}}\right)V\left(v_{m},v_{\text{0}}\right)T(\Delta t) \end{array}$$ (2)

where $c_{\text{0}}$ and $c_{m}$ are concentrations of the reference and measured particle, $v_{\text{0}}$ and $v_{m}$ are the corresponding velocities, and Δt is an offset due to minor differences in how the events are windowed. Thus, the matrices $S\left(R_{m},R_{\text{0}}\right)$, $V\left(v_{m},v_{\text{0}}\right)$, and T(Δt) can be understood as characterizing how changes in size, velocity, and windowing affect the signal shape. See Fig 1.

In principle, the unknown parameters can be determined by solving an optimization problem that minimizes the distance between $\Lambda {\hat{f}}_{m}$ and ${\hat{f}}_{\text{0}}$. In practice, however, it is necessary to regularize (effectively, to set the scale) of $R_{\text{0}}$. We estimate that $R_{\text{0}}$ should be close to the value $R_{\text{0}}\approx \;\frac{\text{2}r}{D_{t}\;}$, where $D_{t}$ is the distance transversed by the particle in the interrogation region and r is the manufacturer specified radius of the particle. $D_{t}$ is calculated from $D_{t}=t_{w}\times v$, where $t_{w}$ is the duration of the signal based on integration bounds as described [9] and v is the average velocity of a particle. We then determine the value of $R_{\text{0}}$ along with the values of $R_{m}$ for 10 additional signals by minimizing the sums of residuals squared of $\Lambda {\hat{f}}_{m}-{\hat{f}}_{\text{0}}$ plus a term proportional to ${\left(R_{\text{0}}-\frac{\text{2}r}{D_{t}}\right)}^{\text{2}}$. We then fix the resulting value of $R_{\text{0}}$, and determine $R_{m}$ and $v_{m}$ for all remaining events by minimizing the sum of residuals squared of $\Lambda {\hat{f}}_{m}-{\hat{f}}_{\text{0}}$ for each remaining signal.

Regarding this technique, several comments are in order. First, it is only possible to determine the relative velocities $v_{m}/v_{\text{0}}$ and relative concentrations $c_{m}/c_{\text{0}}$. However, additional information about the absolute velocity and/or concentration of the reference signal can be used to assign units to $v_{m}$ and $c_{m}$. Second, the analysis represents time-series using a finite number of Fourier modes. In this work, we represent these signals using the first 15 modes, which amounts to applying a low-pass filter to the raw data. This Fourier mode cutoff was selected because it was the beginning of the noise floor for most signals. Finally, only populations that mapped well on to the reference curve were used in the analysis, see the SI Methods and S8 Fig in S1 File for further detail.

## Results and discussion

Fig 1 shows the expected differences in the resulting signal from modification of a particle’s velocity, size, and dye concentration according to this model. In the following sections, we utilize standard reference beads to validate these dependencies and show appropriate relationships based on known population characteristics.

### Selection of the reference pulse for normalization of signals from a population

**Fig 2:**
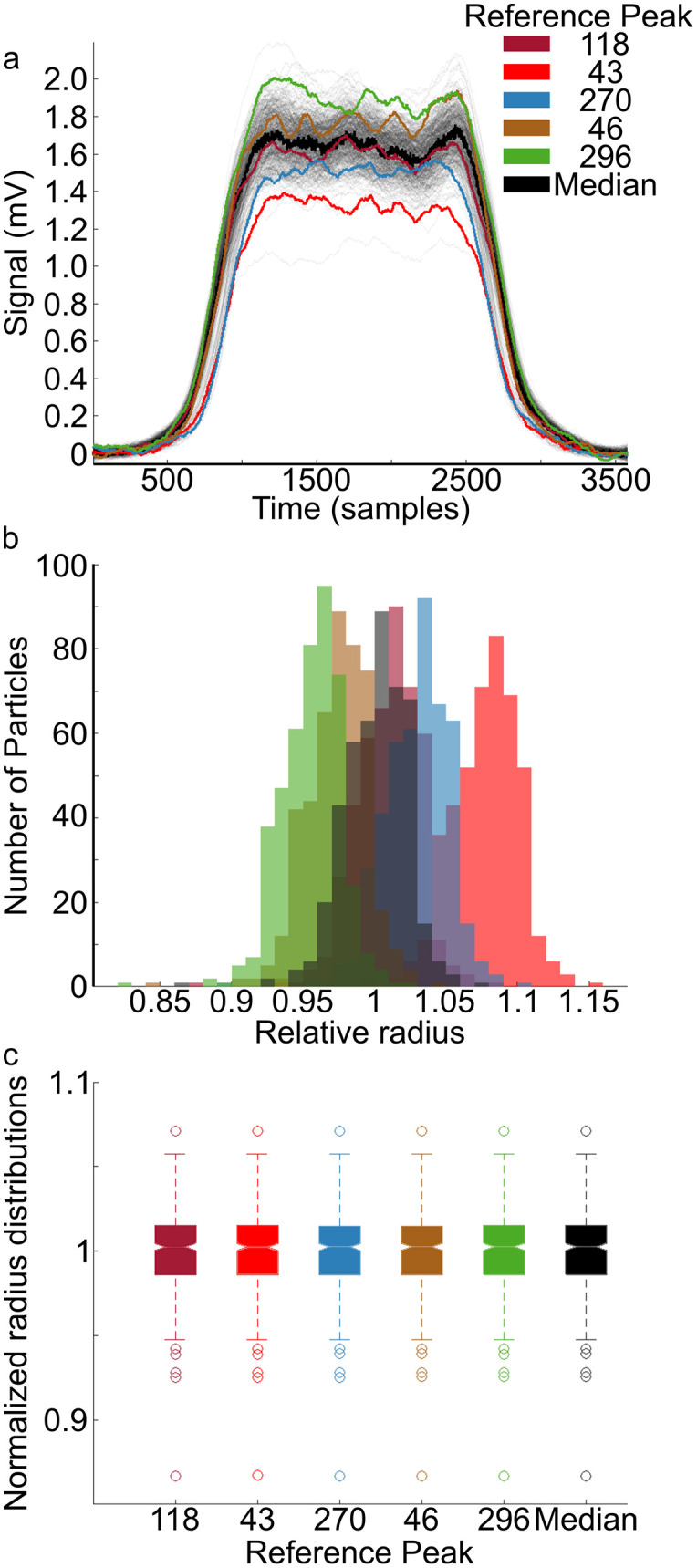
STA analysis based on reference signal choice. a) Overlay of time-aligned, raw time-series of bead measurements. Colored curves indicate various reference signals that were tested. b) The relative radius distributions of the same bead population are overlaid given reference signal choice. c) Box and whisker plots of the radii for each reference signal after normalizing the mean of each distribution to 1. Throughout this figure the median refers to the pointwise median.

We first consider how the choice of reference signal impacts the estimated sizes and velocities (when concentration is assumed fixed). Previously [6], the reference signal was selected to be representative of the population but otherwise random. To test the sensitivity of the analysis with respect to the chosen reference, we estimated the relative radii when using five reference signals spanning a range of amplitudes, as well as the pointwise (in time) median of all the signals. The pointwise median signal was not considered as a reference because, while it is a good estimator of signal scales, it is not guaranteed to retain the shape of the true traces. Comparison of the distributions of the relative radii (Fig 2b) clearly shows a reference-dependent bias. Dimmer particles yield larger relative radii (i.e., primarily $R_{m}/R_{\text{0}}>\text{1}$), while brighter-than-average reference choices resulted in distributions with smaller apparent relative particle radii (primarily $R_{m}/R_{\text{0}}<\text{1}$). The signal (118) closest to the pointwise median signal (Fig 2a, see Methods) resulted in a distribution with mean relative radius of 1.0093 and standard deviation of 0.0223, which was smaller compared to the manufacturer’s specified normalized radius variation of 0.0456. Importantly, however, the differences in the distributions of the relative radii were not statistically significant (by Kruskal-Wallis Test) between all reference choices (p ≈ 1) (Fig 2c). Therefore, for convenience and to abrogate the need for normalization, the reference closest to the pointwise median was utilized for this work.

### Validation of particle size while varying particle velocity in a serial microcytometer

**Fig 3:**
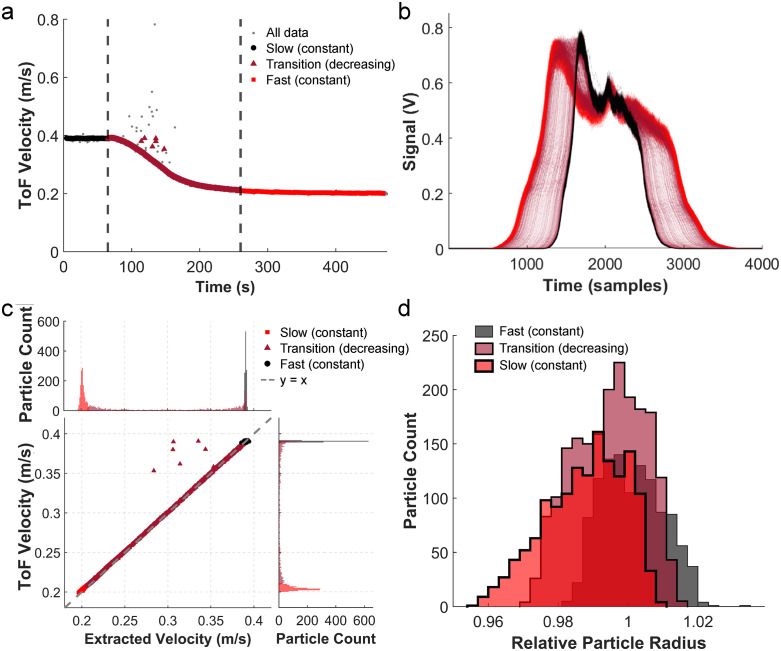
STA analysis of particles having different velocities. **a)** Plot of the velocities of bright, 15 µm diameter particles based on time of flight between two measurement regions over time. Each dot represents a single particle, with the colors representing the clustering of the data. Beads that were collected while the time-averaged velocity was 0.390 m/s (fast) are shown in black, beads collected during transition to slower velocity are shown in maroon, while beads that were collected while the time-averaged velocity was 0.204 m/s (slow) are shown in red. Grey dots indicate that the beads were not used in the analysis. **b)** Overlay of raw signal pulses from beads collected at different total flow rates. **c)** Relative time-averaged velocities versus relative extracted velocities. The gray dashed grey line shows a liner regression with an equation of y = 0.99x + 0.014 and correlation coefficient, R^2^ = 0.999. Histograms of extracted velocities and time-averaged velocities are shown above and to the right of the main figure, respectively. **d)** Overlay of histograms of the relative radii extracted from STA from different conditions. Bin width is to scale on axis.

Our previous study used one stable flow condition for the flow cytometer. Here, to more fully assess the accuracy of velocimetry using STA, a single population of fluorescent beads was measured under controlled conditions in which the particle velocities were dynamically varied between 0.390 m/s to 0.204 m/s. Data was collected on a 3D-flow-focusing serial microcytometer [9] using inertial microfluidics to tightly control particle time-of-flight through a series of laser regions while maintaining a constant sheath-to-core fluid ratio (SCR). The experiment was divided in three ToF velocity regimes (Fig 3a): [1] fast flow (constant), [2] transition flow (decreasing), and [3] slow flow (constant). As expected, decreases in velocity corresponded to a broadening of the duration of the raw signal traces (Fig 3b). Overlaps of signal reconstructions following the STA are shown in S2 Fig in S1 File. The distribution of extracted velocities is shown in Fig 3c. We then compared the time-average velocities from serial cytometry (normalized to the fastest flow condition) to the extracted velocities of the STA method (Fig 3c). When fit to a line the resulting slope (0.99 ± (95% confidence intervals)) indicates a *strong* agreement between the two estimates of velocity. The resulting histograms of the velocities demonstrated a decrease in velocity proportional to the change in flow rate and matched the time-averaged velocity measurement between regions (Fig 3c), while importantly showing no change in estimation of the particle radius (Fig 3d). The relative radii of all populations were centered around 1, which agrees with the particles all having the same diameter.

The serial microcytometer used here [9] could obtain multiple replicates of the particle pulses, which may be used for uncertainty quantification. However, this work focuses on generalizing STA for broad application for flow cytometry without the requirement for serialization. Furthermore, we have already previously shown how comparing serial flow cytometry measurement can help to minimize instrument variation and apparent population spread. Thus, we deem that a study examining the use of measurement replicates to improve shape analysis is out of scope of this work and an opportunity for future metrology innovations.

### Validation of particle size with constant dye concentrations

**Fig 4:**
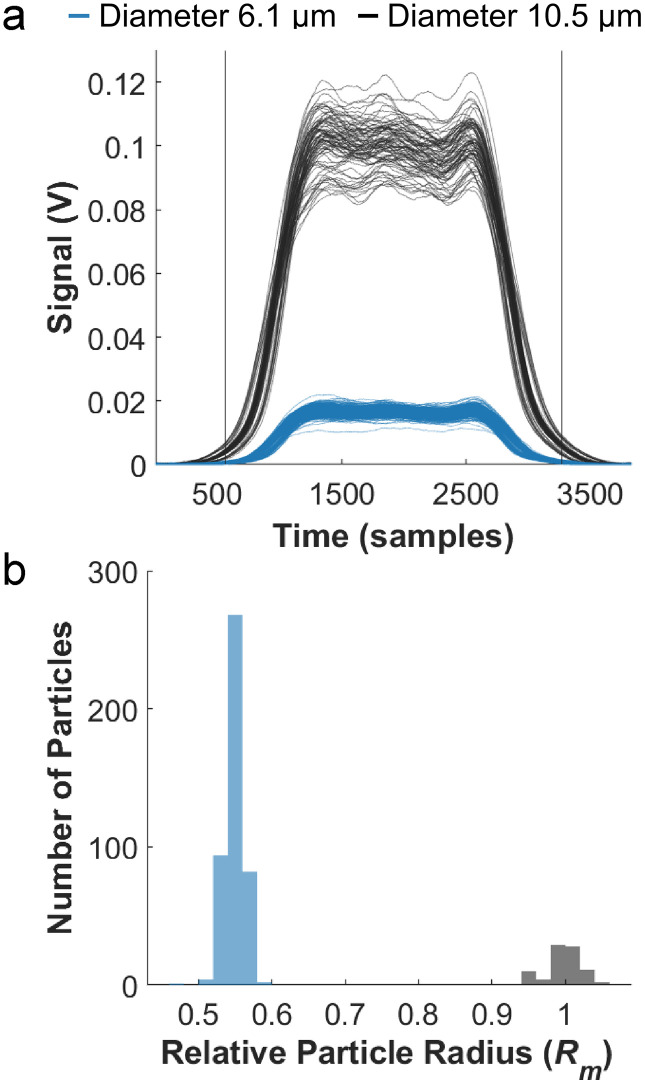
Beads with different sizes but similar dye concentrations were measured and analyzed with the STA. a) Overlays of fluorescent pulses of the selected subpopulations, black indicates the 10.5 µm diameter bead and blue indicates the 6.1 µm diameter bead. b) Histogram of the relative particle radius of the measured beads.

We next verified the STA’s ability to correctly extract size independently from velocity and fluorescence concentration within the particle variation. Two calibrated bead ladders with known fluorescent intensities (specified as mean equivalent fluorescein intensity (MEFL)) and nominal sizes (6.1 μm and 10.5 μm diameters) were used in this test. One particle from each size ladder with similar concentrations was selected. From the 6.1 μm bead set, we chose intensity 5 (1,338 MEFL/µm^3^), and from the 10.5 μm bead set we chose intensity 7 (1,661 MEFL/µm^3^) (see S1 Fig in S1 File and Methods 2.5). Pulses for both subpopulations are shown in Fig 4a. One signal from the 10.5 µm diameter bead population was selected as the reference signal. The data was then processed with the STA. Reconstructions of data from the two populations after STA are shown in S2b Fig in S1 File. The distributions of calculated relative radii for the different subpopulation are shown in Fig 4b. The mean relative radius for the smaller non-reference bead (6.1 µm diameter) bead population was 0.550 ± 0.014 (mean ± standard deviation), which is a 5% difference from the expected relative radius of 0.581 calculated from the manufacturer specified nominal radii. The relative velocities are not different between the two bead populations (S3 Fig in S1 File).

### Validation of particle size with varying dye concentrations

**Fig 5:**
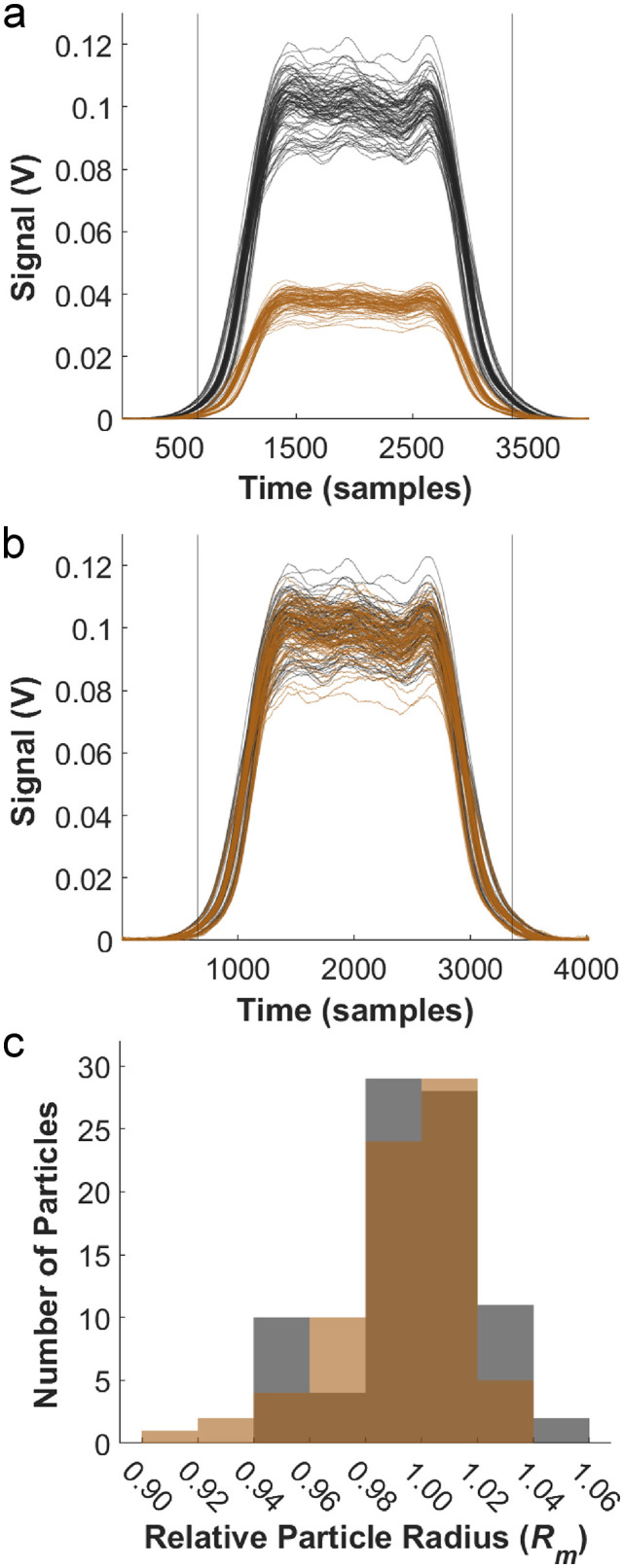
Comparison of beads with different fluorescence intensity values. **a)** Fluorescence pulses of the beads for the two different 10.5 µm bead intensities (MEFL of 381106 (black) and 128924 (brown)). **b)** Overlays of the pulses after applying a correction factor to the population with the smaller MEFL. **c)** Histogram of the two bead populations relative particle radius showing a distribution of similar width.

To complete our validations, beads having the same radius, but different concentrations were compared to one another using the 10.5 μm bead ladder. Bead intensities 7 (1,661 MEFL/µm^3^) and 6 (628 MEFL/µm^3^) were selected for comparison (Fig 5a). The ratio of bead intensity 7 mean pulse height to bead intensity 6 mean pulse height was chosen as the correction factor to normalize fluorescence concentration differences (Fig 5b). Importantly, based on the assumption that intensity of bead is much more strongly influenced by variation in radius than by variation in concentration, the STA routine was set to extract a distribution of radii from the signals without taking into consideration variation in the concentrations of fluorophores within the beads. The resultant distributions of relative radii extracted from STA using beads with different fluorescent dye concentrations were centered on top of one another (Fig 5c). Using a reference signal from near the median height of intensity 7 beads, STA returned mean ± standard deviation relative radii of 0.992 ± 0.030 (intensity 7) and 0.970 ± 0.037 (intensity 6). Overlays of the reference curve and the reconstructed traces following STA are shown in S2c Fig in S1 File. Results based on other intensity combinations are shown in S4 Fig in S1 File. The signal to noise ratio of lower intensity beads results in increased uncertainty in the parameters extracted from the STA reconstruction. There are opportunities to fine-tune STA for specific datasets, for example, to better handle different signal to noise ratios or intensity differences. However, since this work seeks to test the general performance in a range of flow cytometry situations, we leave additional study and optimization of this analysis for the future.

### Applying the model to a biological system

**Fig 6:**
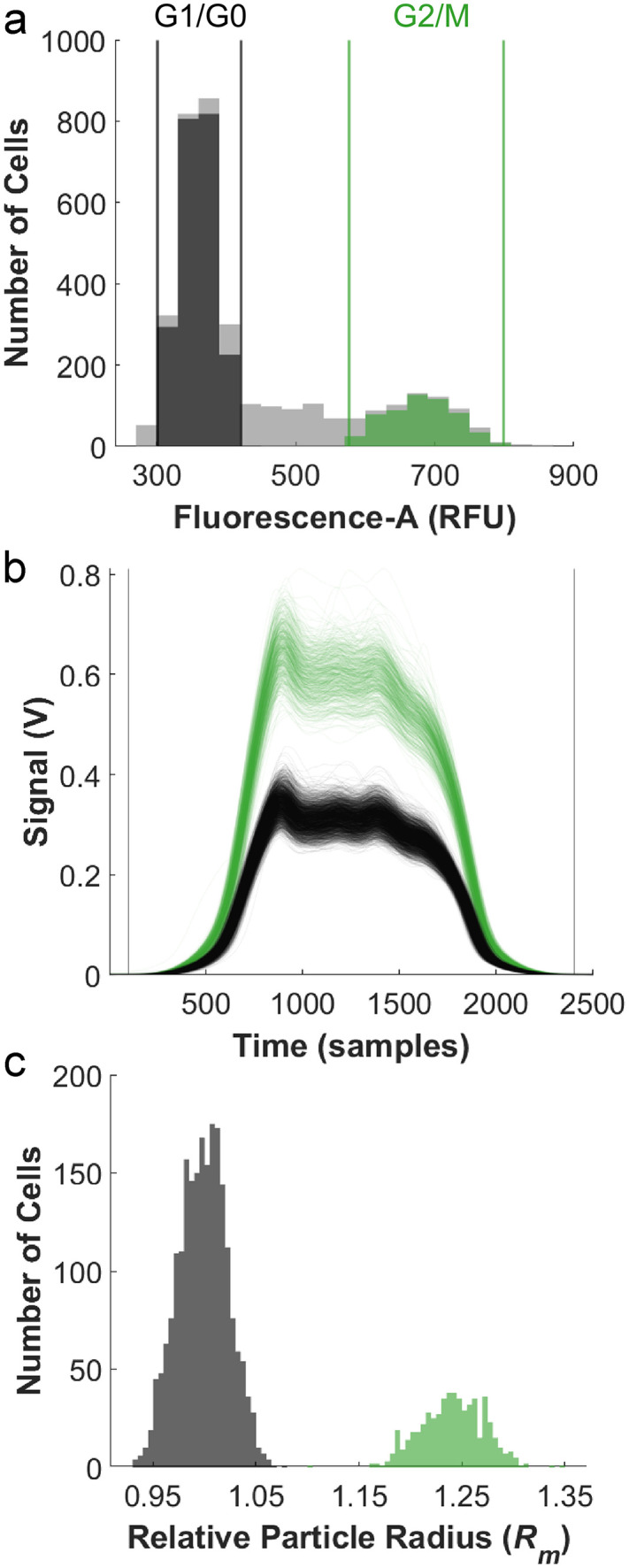
Hoechst 33342 labeled Jurkat cells measured in a serial microcytometer. **a)** Histogram of the fluorescent area used to gate cells. **b)** Raw fluorescence pulses for cells gated from the histogram. **c)** Histogram of nuclei relative radii extracted from STA. Cells likely in G1/G0 (1x DNA) labeled dark gray and cells likely in G2/M (2x DNA) labeled in green.

Next, we demonstrate that the STA can be applied to fluorescently stained biological samples. Jurkat cells were stained with the DNA binding dye Hoechst 33342, which resulted in an expected bimodal distribution of cells that range in DNA content from G0/G1 (1x) to G2/M (2x) (Fig 6a). Cells were gated based on their fluorescence area. A signal from the population of cells that would typically be classified in the G0/G1 (gray) state was used as the reference signal for the STA algorithm. Overlays of pulses for both populations are shown in Fig 6b. The concentration of fluorophore in each population was not normalized and assumed to be consistent between cells in G2/M and cells in G0/G1. As with the STA above, we assume that additional variations in concentration are overshadowed by variations in nuclear radius. Additionally, we assume minimal impact to the signal from variations in cellular granularity, as the optical profile in the microfluidic cytometer is both relatively uniform and considerably wider than a single particle. Overall, this effect amounts to integration (and thus smoothing) of the signal by the detector.

Overlays of the signals collapse onto the reference signal are shown in S5 Fig in S1 File. As expected from time-of-flight measurements, velocities extracted from STA were similar for the cells in different stages of the cell cycle (S6 Fig in S1 File). G2/M cells showed a median nuclear radius increase of 1.26-fold (Fig 6c). This shift in radius corresponds to a 2.0-fold change in the volume, which would be consistent with the nuclear volume doubling after S-phase DNA replication [12]. Additionally, fluorescent imaging of cells shows a similar trend for increased nuclear size in G2 cells compared to G1 cells (S7 Fig in S1 File) with an observed ratio of 1.33.

## Conclusions

We demonstrated for the first time that an STA method can extract relative size differences between subpopulation of fluorescently labeled particles and cell organelles. By using the nuclei of cells as a biological size standard, we may also obviate the need for size standards that do not match the optical properties of the target cells or organelles. Demonstrated here was a single stain for a well-studied biological system, but the principles/methods outlined in this paper can be applied to more complex and relevant biological questions. For example, STA may be used to study differences in cells that are surface labeled versus those stained cytosolically, or to resolve differences in cells exhibiting different deformability phenotypes. There is also more work to be done on optimizing the algorithm, to account for concentration differences. Additionally, there are avenues of research to test how different laser profiles may improve compatibility with the algorithm and reduce uncertainty in the estimation of particle size. Although conventional flow cytometers do not offer the ability to analyze time-series data of signal pulses, it is our hope that highlighting the value of these measurements may encourage instrument manufacturers to offer such measurement capabilities to advance the field.

## Supporting information

S1 File This file contains supporting methods, tables of bead and experimental properties, and supplemental figures. Data files for Figs 3–6 are also available.
(DOCX)

S1 Data This is the.xlsx data file supporting Fig. 3. (XLSX)

S2 Data This is the.xlsx data file supporting Fig. 4. (XLSX)

S3 Data This is the.xlsx data file supporting Fig. 5. (XLSX)

S4 Data This is the.xlsx data file supporting Fig. 6. (XLSX)
