# Supplementary material for: Estimating particle size and velocity from fluorescence pulses: A practical validation study of flow cytometry signals analysis
Source: PLoS One. 2026 May 18;21(5):e0348292. doi: 10.1371/journal.pone.0348292 (PMC13183216; doi:10.1371/journal.pone.0348292)
Supplement: S1 File — Data files for Figs 3–6 are also available. (DOCX) [file pone.0348292.s001.docx]

**Supplemental information**

Data files associated with Figures 3-6 are included as supplemental information.

**Methods**

**Population inclusion metrics**

When comparing samples that include multiple bead populations (e.g., different fluorophore concentrations, sizes, or velocities), residuals of the pointwise median of the reconstructed curves versus pointwise median of the model curves were checked to ensure a good fit by taking a residual between the model and different populations. Populations with approximately centered and symmetric residuals were considered appropriate for comparison. (See **S8 Fig** for an example of residual comparisons).

**Imaging and image analysis**

Cells were stained following the protocol from section 2.2, subsection “sample preparation”. Then aliquots of 200 µL of the cell solution was added to a 96 well plate. Fixed cells were allowed to settle for 1 hr. Fluorescence microscopy images of the plate were acquired on an inverted epifluorescence widefield microscope with LED illumination. A CMOS camera (pixel size of 3.45 μm, frame rate of 20 s^−1^, 14-bit resolution) with a 20 × magnification lens was used to acquire images within the 96 wells. Cells nuclei sizes were quantified using a custom Matlab script. Background in images was subtracted by a top-hat filter, and manual intensity thresholding was used to segment the cells. Finally, blob analysis was used to calculate the equivalent diameter of all segmented objects.

**Supplemental Tables and Figures**

**S1 Table. S1 Table. Properties of beads used for comparison of fluorescence and size.**

| Nominal Diameter (µm) | Ladder number* | MEFL | Concentration (MEFL / µm^3^) |
| --- | --- | --- | --- |
| 6.1 | 5 of 6 | 159 034 | 1338 |
| 10.5 | 7 of 9 | 1 006 897 | 1 661 |
| 10.5 | 6 of 9 | 381 106 | 628 |

*Particle subpopulation number is in order of increasing intensity and includes the blank bead.

**S2 Table.** **Flow rates used for velocity experiments with corresponding particle-based Reynolds numbers, time of flight measured velocities, and CV of the velocity.**

| *Total Flow rates (µL/min)* | *Re_p_^*^* | *Velocity (m/s)* | *Velocity CV (%)* |
| --- | --- | --- | --- |
| *84* | *4.17* | *0.390* | *0.16* |
| *42* | *2.08* | *0.204* | *1.21* |

*^*^* ^Calculated for 15 µm diameter particles^

**
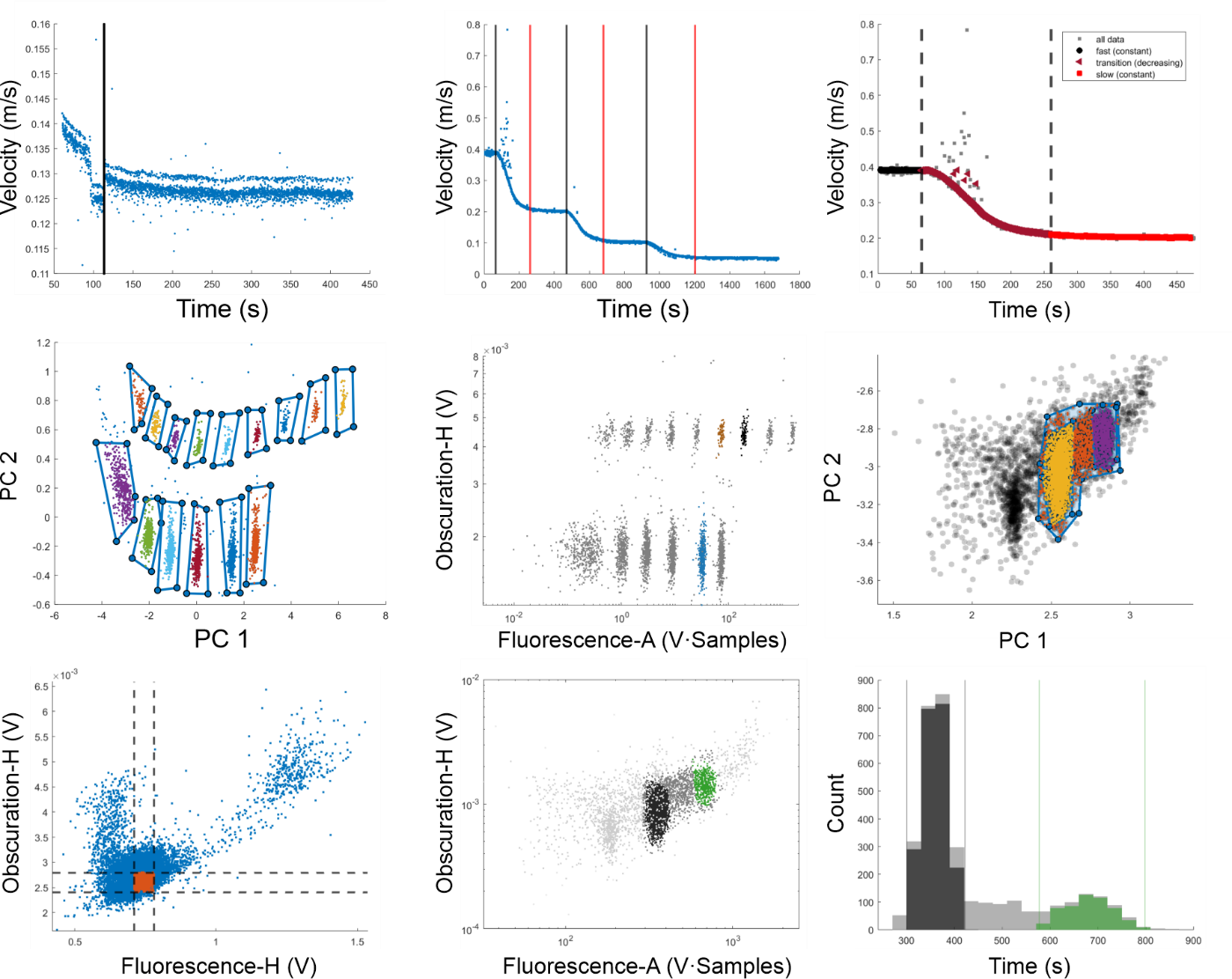
**

**S1 Fig.** **Panels outlining gating strategies.** For many of the studies in this manuscript, beads were gated based on velocity. For example, in the first panel gating included selection of beads with stable velocity, e.g., after 110 s of run time, as indicated by selecting particles to the right of the black line. This panel also shows two distinct velocity populations, which result from two different particle sizes in the sample (e.g., panels 4 and 5). Beads were also gated to analyze specific ranges of velocities, either during periods of stable velocities, such as between red and black lines on the second panel, or between periods of changing velocities, such as between black and red lines on the panel. A more detailed view of one region of the velocity gating is shown in the third panel. To analyze beads of specific size or intensity, additional gates were selected using either principal component analysis (PCA) or conventional fluorescence and scatter channels, as indicated in the fourth through the ninth panel.

**
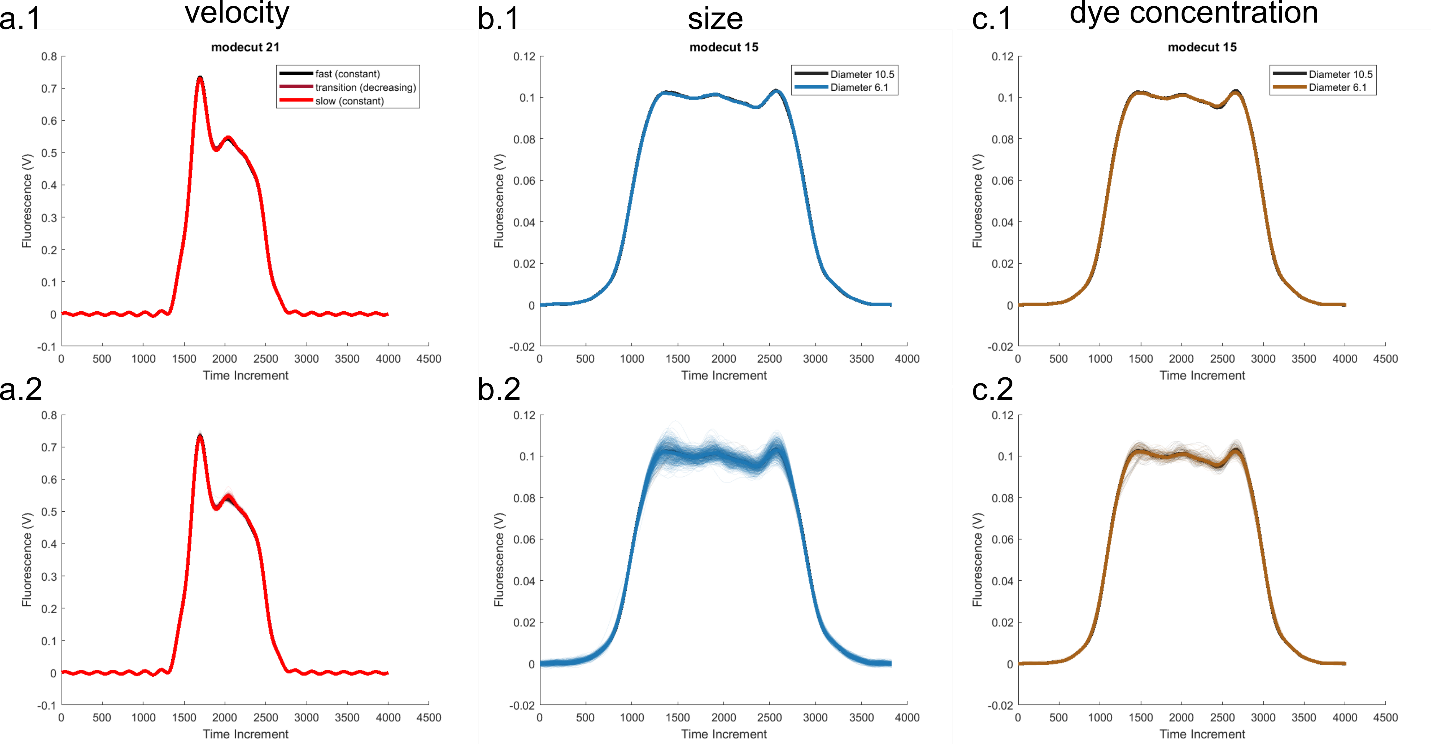
**

**S2 Fig.** **Reconstructed fluorescence curves post STA for the different validation experiments.** Plots show overlaps of fluorescence pulses from beads after STA transformations for conditions with varying a) velocity, b) size, and c) dye concentrations. Top row of plots (.1) indicates pointwise (in time) median of collapsed traces for each subpopulation denoted in the legends. Bottom row of plots (.2) shows all collapsed traces overlaid.

**S3 Fig.** **Histogram of relative velocities for different sized beads in the microcytometer.** Plot shows the count distribution of 10.5 µm diameter beads (blue) and 6.5 µm diameter beads (green) following velocity normalization.


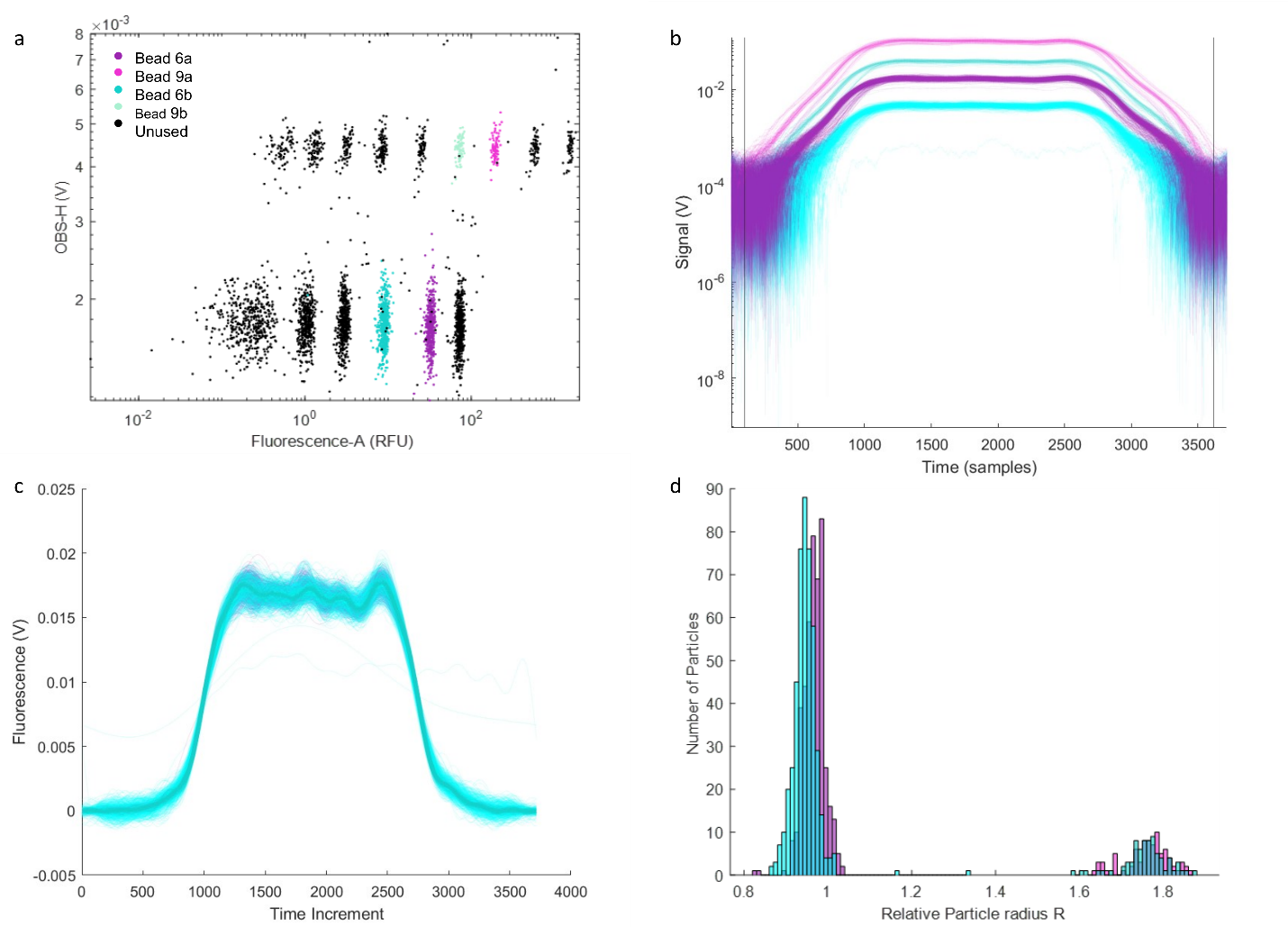


**S4 Fig.** **Multiple size and concentration beads compared using the STA.** a) Result of particle gating; b) Raw signals; c) Collapsed signals; d) Extracted particle size distributions. Beads that are labeled 9a and 6a (pink and purple) have the same concentration of dye. Beads that are labeled 9b and 6b (teal and blue) are a second lower matched concentration of dye pair. Labeled 6 or 9 refers to the concentration ladder number of the bead and beads within the same ladder has the same size.


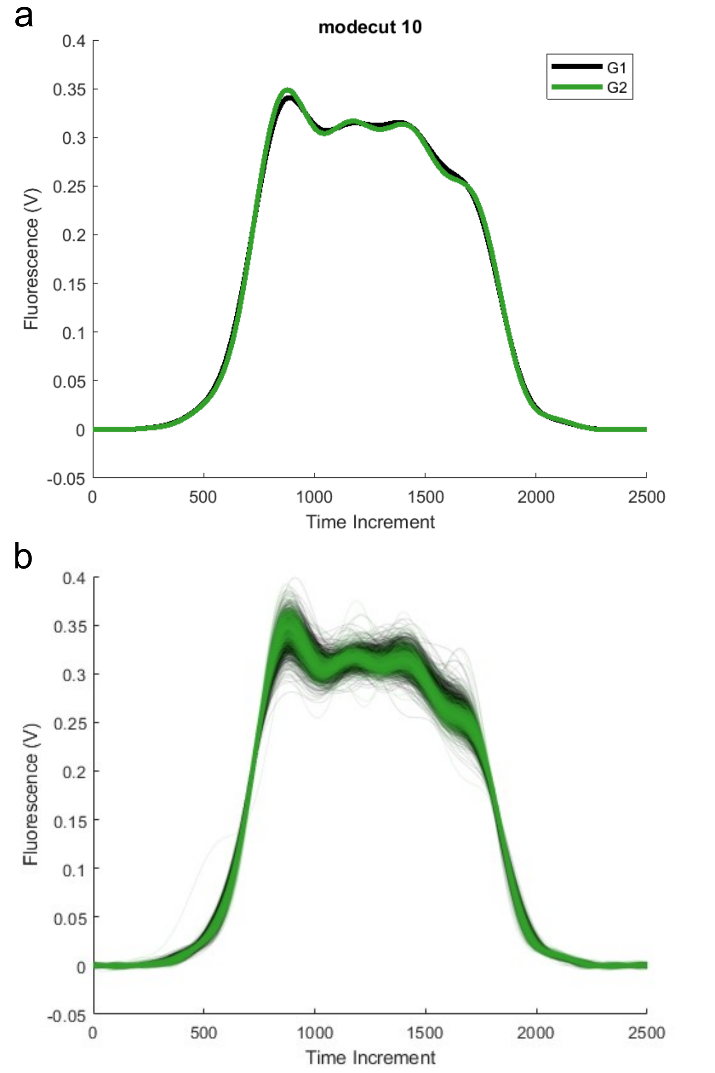


**S5 Fig.** **Reconstructed curves post STA for the comparison of Jurkat cells with different Hoechst 33342 staining intensities, as used to indicate cells in G1 or G2 phase of the cell cycle.** a) Pointwise (in time) median of collapsed traces for each cell subpopulation; b) All collapsed traces.


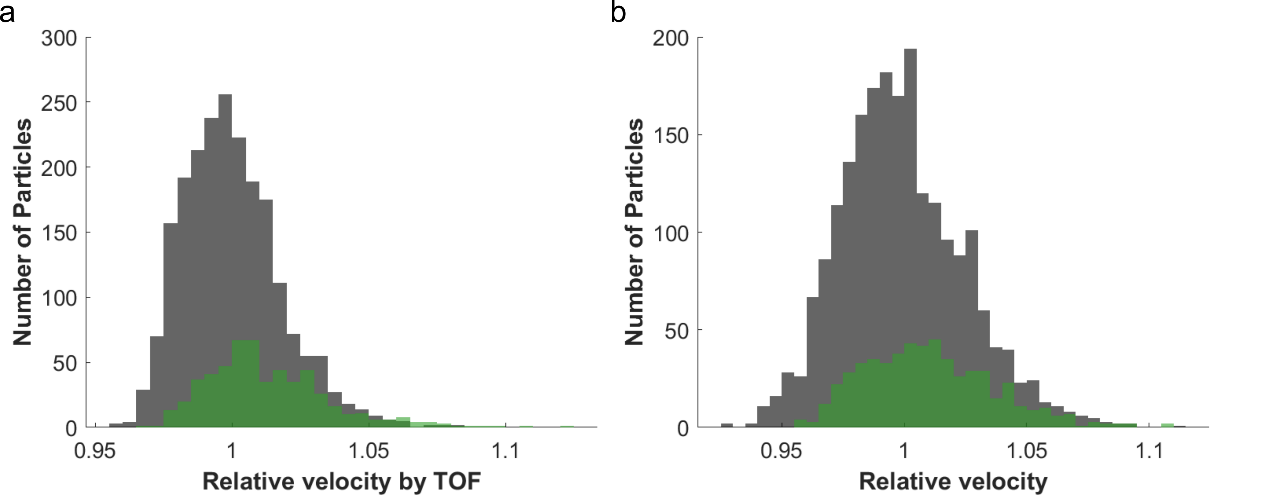


**S6 Fig. Estimates of velocity of cells based on their position in the cell cycle.** Histogram of the relative velocity using ToF (a) and STA (b) for cells gated in G1 (black) and G2 (green).


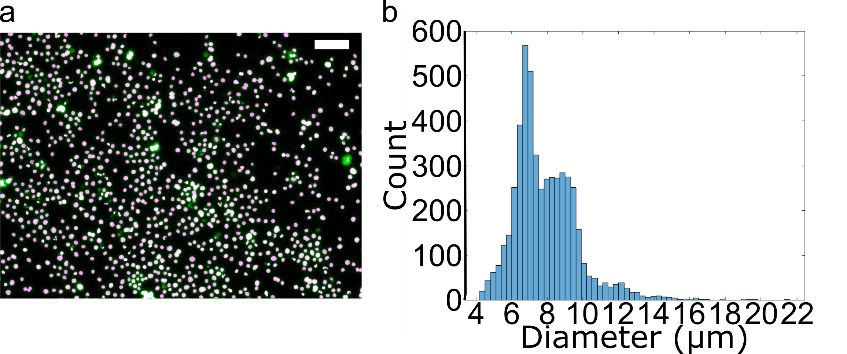


**S7 Fig.** **Nuclear size validation by microscopy.** a) A fluorescent microscopy image of the nuclei of Jurkat cells stained with Hoechst 33342. Fluorescent signals are falsely colored green, while the binary masked that mark the cells is colored purple, and white indicates overlap of both the fluorescence image and the mask. Scale bar is 50 µm. b) A histogram of the equivalent diameters calculated from the binary masks shows two pulses at approximately 6.75 microns and 8.85 microns.


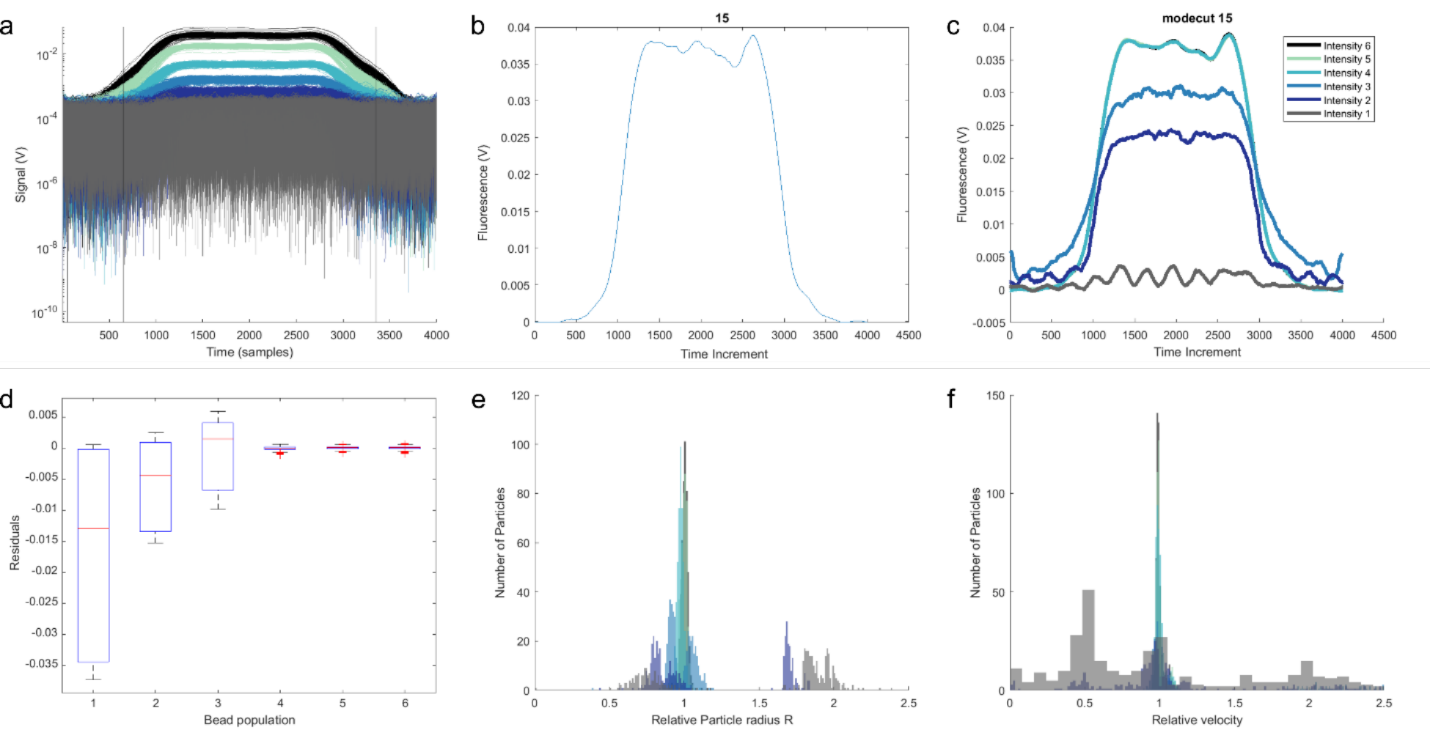


**S8 Fig. Demonstration of residuals check for population inclusion in analysis.** a) Fluorescence curves for 6 different intensity beads labeled with different colors. b) Pointwise (in time) median of the model construction curves used in the analysis. c) Pointwise medians of the reconstructed curves for each bead intensity. Note that the pointwise median traces for beads 4, 5, and 6 are overlapped because they all map well to the reference curve. d) Box plot of the residuals of the pointwise medians of the different intensity beads compared to the median of the model curves. Because beads 1, 2, and 3 have large residuals and do not map well onto the reference curve (see panel c), they are excluded from the analysis. e) Overlay of the histograms for the relative radii extracted by the method. Note that populations that are skewed and whose residuals are not centered around zero (see panel d) also give inaccurate estimates of the radii. f) Overlay of the histograms for the relative velocities extracted by the method. Similarly, populations that are skewed and whose residuals are not centered around zero (see panel d) also give inaccurate estimates of the velocities.
